# Relaxant Effect of Monoterpene (−)-Carveol on Isolated Human Umbilical Cord Arteries and the Involvement of Ion Channels

**DOI:** 10.3390/molecules25112681

**Published:** 2020-06-09

**Authors:** Renata Evaristo Rodrigues da Silva, Andressa de Alencar Silva, Luís Pereira-de-Morais, Nayane de Sousa Almeida, Marcello Iriti, Marta Regina Kerntopf, Irwin Rose Alencar de Menezes, Henrique Douglas Melo Coutinho, Roseli Barbosa

**Affiliations:** 1 Department of Biological Chemistry, Regional University of Cariri, Crato 63105-000, CE, Brazil; renata_ers@hotmail.com (R.E.R.d.S.); nayanealmeida1@gmail.com (N.d.S.A.); martaluiz@yahoo.com.br (M.R.K.); irwinalencar@yahoo.com.br (I.R.A.d.M.); hdmcoutinho@gmail.com (H.D.M.C.); roselibarbo@gmail.com (R.B.); 2 PhD student Graduate Program in Physiological Sciences, Higher Institute of Biomedical Sciences State University of Ceará–UECE, Fortaleza 60714-903, CE, Brazil; andressaalencar17@hotmail.com; 3 PhD student in Biotechnology by the Northeastern Biotechnology Network - RENORBIO, State University of Ceará-UECE, Fortaleza 60714-903, CE, Brazil; luispereira256@gmail.com; 4 Department of Agricultural and Environmental Sciences, Milan State University, via G. Celoria 2, 20133 Milan, Italy

**Keywords:** carveol, human umbilical artery, vasorelaxant

## Abstract

Carveol is a monoterpene present in the structure of many plant products. It has a variety of biological activities: antioxidant, anticancer and vasorelaxation. However, studies investigating the effect of monoterpenoids on human vessels have not yet been described. Thus, the present study aimed to characterize the effect of (−)-carveol on human umbilical arteries (HUAs). HUA ring preparations were isolated and subjected to isometric tension recordings of umbilical artery smooth muscle contractions. (−)-Carveol exhibited a significant vasorelaxant effect on KCl and 5-HT-induced contractions, obtaining EC_50_ values of 344.25 ± 8.4 and 175.82 ± 4.05 µM, respectively. The participation of calcium channels in the relaxation produced by (−)-carveol was analyzed using vessels pre-incubated with (−)-carveol (2000 µM) in a calcium-free medium, where the induction of contractions was abolished. The vasorelaxant effect of (−)-carveol on HUAs was reduced by tetraethylammonium (TEA), which increased the (−)-carveol EC_50_ to 484.87 ± 6.55 µM. The present study revealed that (−)-carveol possesses a vasorelaxant activity in HUAs, which was dependent on the opening of calcium and potassium channels. These results pave the way for further studies involving the use of monoterpenoids for the vasodilatation of HUAs. These molecules have the potential to treat diseases such as pre-eclampsia, which is characterized by resistance in umbilical arteries.

## 1. Introduction

Over the years, science has shown that natural products are a source of numerous substances with different chemical structures, which can be used in different biological studies [1]. Plants are capable of producing secondary bioactive metabolites that have great potential to interact with biological molecules. Thus, secondary metabolites facilitate the development of drugs and the identification and characterization of new cellular targets, as well as provide an opportunity to elucidate the mechanism of action of these molecular interactions [2]. Within the identified secondary plant metabolites are terpenoids, which constitute the largest secondary metabolite group. Importantly, they have been shown to be bioactive in different animal models [3].

The monoterpene carveol is a molecule widely used in the perfume, soap and shampoo industries [4]. Carveol has also been shown to be an interesting compound with pharmacological activities, which include a repellent effect when used against *Anopheles gambiae* [5], a nematocidal action against *Meloidogyne incognita* [6] and antibacterial activity [7]. Importantly, it has a low toxicity profile [8]. Moreover, carveol administration to the diet of rats with breast cancer has shown efficacy in combating early-stage cancer [9]. Additionally, an anti-inflammatory activity [10] and a prolongation of anesthetic effects [11] have also been demonstrated for carveol. Carveol has also shown a myorelaxant effect in isolated rat aorta sections in smooth muscle studies [12]. However, vasorelaxant effects have not been described in human tissues.

Umbilical cords and their derivatives have been the targets of important research, such as in models for cardiovascular disease studies [13], in tissue engineering and regenerative medicine [14], as well as for investigating new substances with relaxant properties [15]. The umbilical cord, often classified as biological waste, presents a range of possibilities for investigating these properties, standing out as an excellent model for the discovery of vasoactive substances [16], in addition to being a biological sample that is easy to obtain and that does not harm the mother nor the fetus [17].

Hypertensive syndromes in gestating women still portray a public health problem, with current treatment for pre-eclampsia generating a lot of discussion, especially when the mother–fetus binomial and its risks and benefits are taken into account [18]. While there are some drugs available for the antihypertensive treatment of gestating women, such as alpha-methyldopa, propranolol, hydralazine and nifedipine [19], the hypotensive treatment for this pathology still remains uncertain [20]. Investigating how new substances may be involved in this process can reveal great therapeutic value in the treatment of pathologies, such as pre-eclampsia [21]. Thus, there exists a growing need for further studies on the application and discovery of vasodilatory compounds. Few clinical studies have analyzed the effects of natural products on the smooth muscle of human umbilical arteries (HUAs). This study aims to investigate the effect of (−)-carveol on HUAs and to evaluate its possible vascular effects, such as HUA vascular contractility relaxation. Such studies may bring new perspectives for the therapeutic treatment of HUA vascular disorders, such as pre-eclampsia.

## 2. Experimental Section

### 2.1. Solutions and Drugs

The drugs and reagents used were of analytical purity, obtained from Sigma Chemical Corporation (St. Louis, MO, USA), stored in accordance with the manufacturer’s instructions. The following salts were used: potassium chloride (KCl), sodium chloride (NaCl), magnesium sulfate (MgSO_4_), calcium chloride (CaCl_2_), glucose (C_6_H_12_O_6_), potassium phosphate (KH_2_PO_4_), sodium carbonate (NaHCO_3_), barium chloride (BaCl_2_), ethylenediaminetetraacetic acid (EDTA), -[4-(2-hydroxyethyl)-1-piperazinyl]ethanesulfonic acid (HEPES) and the solubilizing agent Tween 80 (CAS Number 9005-65-6). The concentrations were expressed in millimole/liter (mM).

Substances such as serotonin (5-HT) and tetraethylammonium (TEA) were dissolved in distilled water, while nifedipine was diluted in ethanol. The (−)-carveol was diluted in distilled water and 3% Tween 80, at a temperature of ±22 °C, and stored with an initial concentration of 1 M. The solutions obtained were stored at 0 to 4 °C and thawed at the time of the experiment.

### 2.2. Tissue Preparation and Isolation

Sample collection and processing were approved by the Human Research Ethics Committee from the Regional University of Cariri (Comitê de Ética em Pesquisa Humana da Universidade Regional do Cariri—URCA, Crato, CE, Brazil, no. 3.832.881) and by the São Francisco de Assis Hospital and Maternity Ethics Committee (Comitê de ética do Hospital e Maternidade São Francisco de Assis, Crato, CE, Brazil). Human umbilical cord fragments (portions that would be destined for biological disposal) of approximately 10 cm were obtained with the consent of the donor mothers, who were healthy, normotensive and without any disturbances in their cord, following vaginal or cesarean delivery. Samples were collected and stored in modified Krebs solution (mM): NaCl 125; KCl 4.8; CaCl_2_ 1; MgSO_4_ 1.2; NaHCO_3_ 25; KH_2_PO_4_ 1.2; C_6_H_12_O_6_ 11; HEPES 25; EDTA 0.3), refrigerated in a thermal box and transported to the URCA Excitable Cell Physiopharmacology Laboratory (Laboratório de Fisiofarmacologia das Células Excitáveis da URCA). The cord segments were stored in a refrigerator at 4 to 8 °C and were used within 48 h after the collection period [22]. The HUA was isolated from its connective tissue and cut into 3 to 4 mm rings. The vascular endothelium was mechanically removed with a cotton thread that was passed through the lumen of the artery to avoid interference from substances released by endothelial cells.

### 2.3. Determination of the Tension Exerted on the HUA Rings

The HUA rings were cleaned of Wharton’s jelly and of connective and adipose tissues. Isometric tension recordings from the HUA rings held in a thermostatic organ bath apparatus were measured using a rod connected to a force transducer (MLT0420). The transducer was connected to an amplifier (ADInstruments Bridge Amps), which was subsequently connected to an analog digital converter platform (BCN/pod port) installed on a computer. The collected data were converted into traces and stored in files using the LabChart Pro software for further analysis.

The rings were individually suspended with stainless steel hooks inserted into their lumens, with an isometric tension of 3 g. This assembly was performed in glass chambers with 10 mL of Krebs–Henseleit solution at 37 °C, with constant bubbling with a carbogenic mixture (95% O_2_; 5% CO_2_). The solution was renewed every 15 min after the artery rings were suspended.

**Figure 1:**
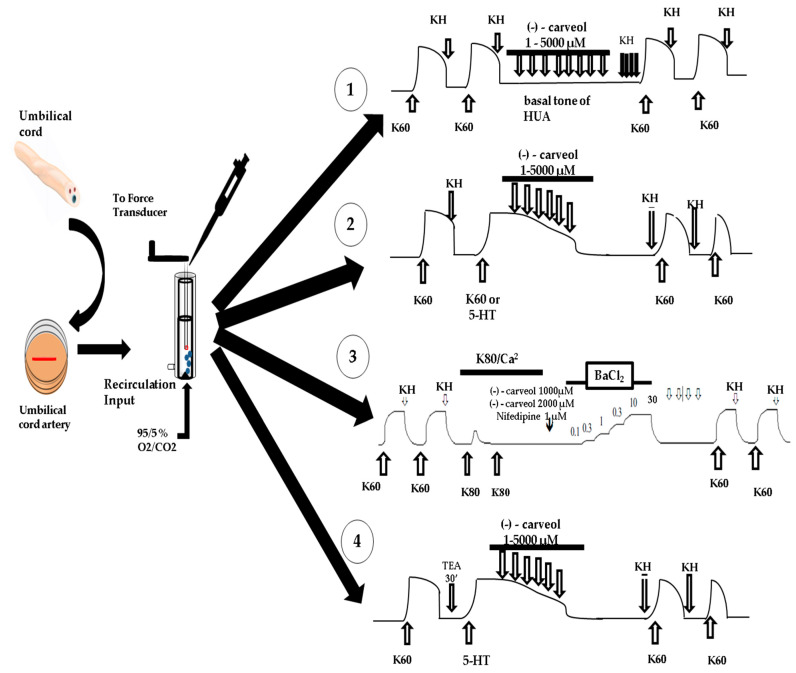
Illustrative scheme of experimental protocols. **(1)** Schematic representation of the effect of (−)-carveol on the basal tone of the HUA. **(2)** Schematic representation of the effect of (−)-carveol on contractions sustained by K60 or 5-HT. **(3)** Schematic representation of the effect of (−)-carveol on BaCl_2_ contractions in the presence of K80 without Ca^+2^. **(4)** Schematic representation of the effect of (−)-carveol on sustained contractions induced by 5-HT in the presence of potassium channel blockers (tetraethylammonium—TEA). (* KH = Krebs–Henseleit solution).

After a stabilization period of approximately 2.5 h, all protocols began with two subsequent contractions, produced by the addition of 60 mM KCl (K60) to the studied HUA rings, in a hypertonic manner, where the maximum response obtained after a plateau was reached—this was considered the maximum contraction of the ring. Only experiments with reproducible contractions were considered viable for the experimental series (Figure 1). Thereafter, the contractile agonists KCl (60 mM) or 5-HT (10 μM) were added to the preparations, followed by the increasing and cumulative addition of (−)-carveol (1–5000 µM) (Figure 1). Sufficient time was allowed for the response to reach a plateau, usually 5 to 15 min, for each new (−)-carveol concentration.

### 2.4. Statistical Analysis

Data are expressed as the mean ± SEM. The Sigma Plot version 11.0 (Systat Softaware- San Jose, CA 95110, U.S.A.) software was used for statistical analysis and graphical production. Results considered statistically significant had a null hypothesis probability of less than 5% (*p* < 0.05). Student’s t-tests and analysis of variance (one- or two-way ANOVA), followed by Bonferroni and Holm–Sidak t-tests, were performed where appropriate. EC_50_ values were determined as the substance concentration capable of producing 50% inhibition of the maximum contraction. A logarithmic interpolation was performed for each experiment in the calculations. Where this was not possible, a linear relationship between two EC_50_ points was obtained.

## 3. Results

**Figure 2:**
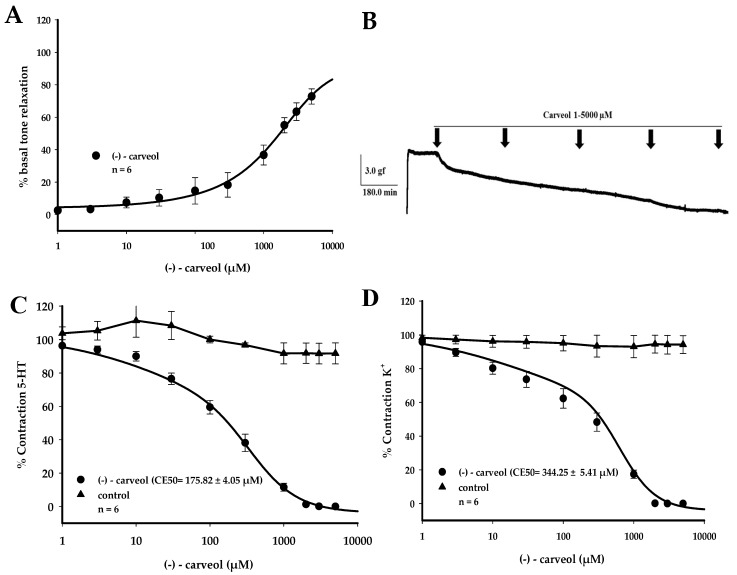
Relaxant effect of (−)-carveol in the HUA on contractions sustained by 5-HT and KCL (60 mM). (**A**) Representative graph of the effect of (−)-carveol on the HUA basal tone. (**B**) Original layout showing the relaxant effect of (−)-carveol (1–5000 µM) on the spontaneous basal tone in the HUA. (**C**) Representative graph of the effect of (−)-carveol on contractions sustained by 5-HT (10 µM). (**D**) Representative graph of the effect of (−)-carveol on contractions sustained by KCl (60 mM). Values are expressed as the mean ± SEM; *n* = 6 (*p* < 0.05, one-way ANOVA followed by Holm–Sidak).

To assess the effect of (−)-carveol on the basal tone of the HUA, increasing and cumulative concentrations of the monoterpene (1–5000 µM) were added to HUA preparations to obtain a concentration–response curve. In these preparations, (−)-carveol reduced the HUA basal tone by up to 72.77 ± 4.69% (*p* < 0.05) (Figure 2A,B). In order to investigate whether (−)-carveol affected the pharmacomechanical excitation-contraction couplig (ECC), HUA smooth muscle contractions were induced with 10 µM of serotonin (5-HT), a potent vasoconstrictor, which activates 5-HT_1B/D_ and 5-HT_2A_ receptors [23]. Increasing and cumulative concentrations of (−)-carveol (1–5000 µM) were added during the tonic phase of the 5-HT contraction, after reaching a plateau, where this induced vasorelaxations in the HUA rings in a concentration-dependent manner. (−)-Carveol produced a statistically significant relaxant effect from the 10 µM concentration, with an EC_50_ of 175.82 ± 4.05 µM (*p* < 0.05, one-way ANOVA, followed by Holm–Sidak; Figure 2C).

After investigation of the effect of (−)-carveol on the pharmacomechanical ECC, its effect on the electromechanical ECC was then evaluated, where HUA smooth muscle contractions were induced with Krebs–Henseleit solution modified with 60 mM KCl [23]. (−)-Carveol induced vasodilation in HUA pre-contracted rings in a concentration-dependent manner; this relaxation was statistically significant from 100 µM. In these conditions, (−)-carveol presented a rightward shift in the curve, increasing the EC_50_ to 344.25 ± 5.41 µM (*p* < 0.05, one-way ANOVA, followed by Holm–Sidak; Figure 2 D). This demonstrates that (−)-carveol was less potent at relaxing rings pre-contracted with KCL than those pre-contracted with 5-HT, in which a greater potency (EC_50_ of 175.82 ± 4.05 µM) was observed, with this difference being statistically significant (*p* < 0.001, one-way ANOVA, followed by Holm–Sidak). 

**Figure 3:**
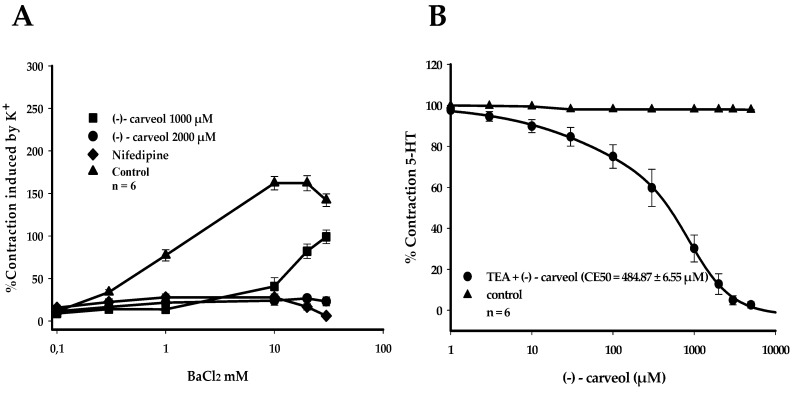
Relaxant effect of (−)-carveol on HUA and evaluation of the participation of voltage-operated calcium channels (VOCCs) and large conductance Ca^2+^ activated K^+^ channels (BKCa). (**A**) Effect of (−)-carveol (1000 and 2000 µM) on contractions evoked by exogenous BaCl_2_, where nifedipine (1 μM) was used as a positive control. (**B**) Representative graph of the effect of (−)-carveol on contractions sustained by 5-HT (10 µM) in HUA sections pre-incubated with TEA (10 mM). Values are expressed as the mean ± SEM; *n* = 6 (*p* < 0.05, one-way ANOVA followed by Holm–Sidak). Please correct the unit in the Figure 3B.

To investigate the involvement of L-type voltage-operated calcium channels (VOCCs) in the relaxation produced by (−)-carveol, experiments were carried out where HUA rings were depolarized in a calcium-free medium in the presence of a supra-maximum concentration of K^+^ (80 mM K^+^). In the absence of (−)-carveol, BaCl_2_ induced contractions in a concentration-dependent manner (Figure 3A), since in the absence of calcium, barium permeates the calcium channel better. These contractions reached a maximum value with 30 mM Ba^+2^ in the control preparation.

In preparations pre-incubated with 2000 µM of (−)-carveol, a complete blockage of contractions in HUA preparations occurred, a behavior similar to that of nifedipine (1 µM), a selective L-type Ca^2+^ channel blocker, which did not show a statistically significant contraction with the 30 mM Ba^+2^ concentration (*p* < 0.05, one-way ANOVA, followed by Holm–Sidak). In this same experimental series, another (−)-carveol concentration (1000 µM) was evaluated; however, this was unable to inhibit the contractions caused by increasing concentrations of Ba^+2^ (Figure 3A).

To investigate the participation of Ca^2+^ activated large conductance potassium channels (BKCa) in the vasorelaxant response promoted by (−)-carveol, 10 mM of tetraethylammonium (TEA) was added to the preparation and incubated for 30 min, followed by a contraction induced with 10 µM of 5-HT. Increasing concentrations of (−)-carveol (1–5000 µM) were cumulatively added to obtain a concentration–response curve, where a change in the EC_50_ was observed, presenting a value of 484.87 ± 6.55 µM (*p* < 0.05, one-way ANOVA, followed by Holm–Sidak; Figure 3B). Moreover, the observed EC_50_ was greater than in the absence of TEA, with this difference being statistically significant (*p* < 0.001, one-way ANOVA, followed by Holm–Sidak), thus demonstrating a partial participation of voltage-dependent potassium channels.

## 4. Discussion

The HUA contractile mechanism is conditioned mostly by the release of local vasoactive substances that regulate umbilical blood flow. These substances include serotonin (5-HT), histamine (His), thromboxane and ions, such as calcium (Ca^2+^) and potassium (K^+^). These substances regulate the activation of receptors or ion channels, which involve mechanisms dependent on and/or independent of Ca^2+^ to elicit a contractile response [23]. Studies contributing to the understanding of the mechanisms involved in HUA contractility are of great pharmacological and physiological value, since this vessel is extremely important for the exchange of gases and nutrients between the fetus and the placenta. In this respect, studies investigating the action of natural products on HUA contractile mechanisms are scarce.

Carveol, a natural monoterpene used in increasing and cumulative concentrations of 1 to 5000 μM, altered the HUA tone in a relaxant manner, demonstrating a vasorelaxant effect in the presence of contractions evoked by 5-HT and K^+^, as well as demonstrating the involvement of L-type Ca^2+^ channels as well as the partial contribution of voltage-dependent K^+^ channels. These results also corroborate the vasorelaxant effects of carveol observed in rat aortas [12].

It is known that the contractile response of smooth muscle cells depends on the increase and maintenance of [Ca^2+^]^I^, through the influx of calcium from the extracellular medium, or from the efflux from intracellular stores. Increases in [Ca^2+^]^i^ can be triggered by transmembrane potential alterations, which can arise from an increased concentration of K^+^ ions in the extracellular medium, resulting in membrane depolarization through the extracellular influx of Ca^2+^ via the opening of voltage-dependent Ca^2+^ channels (electromechanical coupling) [24]. Elevation of [Ca^2+^]^i^ can also occur through the binding of external agonists such as 5-HT or His, including others, which trigger a signaling mechanism involving the activation of a complex cascade of secondary messengers. These include IP_3_ and diacyl glycerol which activate internal receptors on the sarcoplasmic reticulum, which contain intracellular Ca^2+^ stores (pharmacomechanical coupling) [25,26].

The pharmacological potency of (−)-carveolin HUAs was statistically greater in the presence of contractions evoked by 5-HT than in contractions evoked by K^+^ (KCl 60 mM), which is reflected by the EC_50_ values for (−)-carveolin and the presence of 5-HT (175.8 ± 4.0 μM) and K+ (344.2 ± 5.4 μM). The greater pharmacological potency of (−)-carveol over serotonergic receptors is of great pharmacological value, since 5-HT is the most potent HUA vasoconstrictive agent, as well as the most common vasoconstrictor used to evoke contractions in HUA vasoactive mechanistic studies [23].

5-HT_2A_ receptors together with 5-HT_1B_/5-HT_1D_ activation are primarily responsible for the contractile effects of 5-HT. The 5-HT_2A_ receptor is coupled to G_q_ proteins and activates phospholipase C resulting in increased levels of the IP_3_ inositol, while 5-HT_1B_ and 5-HT_1D_ receptors are coupled to adenyl cyclase, the G_i/0_ protein inhibitor [26,27].

Our data differ from what has been found for carveol on rat aorta, where a greater pharmacological potency was observed in the presence of K^+^ [12]. However, it is noteworthy that differences in response can occur both between vessels from experimental animals (rodents) and humans, which may be due to distinctions between them in terms of physiology, types of receptors and the action of certain signaling pathways [15,23,28]. Given that studies suggest little participation of endothelium-dependent factors in the vasorelaxant effect of HUAs [28], only preparations from Cardoso-Teixeira et al. [12], which were devoid of an endothelium, were used as a comparative value where the effect of carveol on HUAs had been previously evaluated.

(−)-Carveol administration altered the baseline vascular tone values sustained in HUA vessels. This may be important because some authors report that an elevated basal tone, associated with a greater vasoconstriction of umbilical vessels, may be associated with vascular changes, such as pre-eclampsia and delayed uterine growth, wherein an effective vasodilator improves umbilical blood flow. It is also known that this umbilical arterial system responds poorly to relaxant agents, such as acetylcholine, sodium nitrite and adenosine, despite these agents being shown to be effective in other systemic arterial systems [28,29,30]. Thus, the present (−)-carveol data on the HUA basal tone indicate that this plant derivative is an effective vasodilatory reagent.

It is also worth noting the pharmacological potency of (−)-carveol observed differs from those reported in the literature, where the present EC_50_ values are lower. Moreover, other studies using monoterpenes in animal vessels, such as citral [31] and carvacrol [32], obtained EC_50_ values greater than those found for (−)-carveol in HUAs. Other studies that evaluated the effect of several endogenous substances in HUAs also revealed higher EC_50_ values than those found in the present study.Perusquía et al. [33] evaluated vasorelaxations mediated by the steroid hormones progesterone and 5β-pregnanediol in human umbilical arteries, reporting EC_50_ values of 276.7 and 933.7 µM, respectively. In summary, the present results demonstrate a greater pharmacological potency for (−)-carveol in HUA vessels than in animal vessels, when compared to other monoterpene animal studies, as well as in comparison to endogenous HUA substances.

Studies using natural products in human umbilical vessels are scarce. Studies demonstrating a vasorelaxant effect in HUAs include Campos et al. [34], which used the polyphenol rich fraction (chlorogenic acid, isoorientin and swertiajaponin) from the *Cymbopogon citratus* extract; Lorigo et al. [35], which used octyl methoxycinnamate, an organic compound used in the manufacture of sunscreens; Speroni et al. [36], which used genistein, a natural phytoestrogen belonging to the isoflavone group; and Massaro et al. [37], which used bee propolis, which induced a vasorelaxant effect in HUAs in the presence of K^+^ (60 mM).

We investigated the involvement of membrane ionic channels in the muscle relaxation caused by (−)-carveol in HUAs. An inhibitory effect on L-type Ca^2+^ channels was found, especially at 2000 μM. Preparations containing (−)-carveol (1000 and 2000 μM) were compared to control preparations incubated with nifedipine and to preparations using BaCl_2_, a L-type Ca^2+^ channel blocker and a L-type Ca^2+^ agonist, respectively. In comparison with the control, it was found that (−)-carveol promotes an inhibitory effect similar to that of nifedipine, and (−)-carveol inhibited the contractions promoted by BaCl_2_.

Corroborating with our findings, Cardoso-Teixeira et al. [12] demonstrated that carveol promoted relaxation in rat aortic preparations that were pre-contracted with BayK-8644. This suggests that this monoterpene may be acting by inhibition of L-type Ca^2+^ channels, since BayK-8644 is an agonist in L-type Ca^2+^ channels [38]. Within the different types of Ca^2+^ channels, L-type VOCCs are sensitive to dihydropyridines, and they are the most commonly studied channels in smooth muscle cells [39,40]. Studies performed by Salemme et al. [41] demonstrated that 1 µM nifedipine antagonizes L-type Ca^2+^ channels, verifying that nifedipine induced a rapid and complete inhibition of Ca^2+^ currents.

Similar to our results, several monoterpenes have been suggested to block VOCCs in smooth muscles from various rat organs; these include citral in the aorta [31], 1,8-cineole, citral and limonene in the trachea [42,43], as well as citral and limonene in the uterus [44]. In addition, endogenous substances, such as progesterone and 5β-pregnanediol, have shown that VOCC inhibition is the main vascular relaxation route stimulated by these hormones in HUA vessels [33]. Other aforementioned natural products, such as octyl methoxycinnamate [35] and genistein [36], have also demonstrated a relaxant effect on HUAs through the inhibition of VOCCs.

The above cited data corroborate those found with HUAs in the present study. Together, they demonstrate the relaxant efficacy of numerous substances, including natural products such as (−)-carveol. They all act on the vasculature through their involvement with L-type Ca^2+^ channels. In addition, the data obtained from (−)-carveol in the presence of elevated K^+^ also support this evidence, since the contraction induced by potassium chloride (KCl) is mainly due to the influx of extracellular Ca^2+^ through VOCCs, where this effect is partially inhibited by specific L-type Ca^2+^ channel antagonists (LTCC) [26].

K^+^ channels play an important role in vascular contractility. The increased (−)-carveol EC_50_ in the presence of tetraethylammonium (TEA) suggests an involvement of (−)-carveol with large conductance Ca^2+^ activated K^+^ channels (BKCa), since TEA is a known inhibitor of BKCa channels in vascular smooth muscle cells [40]. Experiments performed by Milesi et al. [45] demonstrated the expression of BKCa channel types in HUA cells, and they showed that TEA completely blocked BKCa channels.

The mechanisms used herein to evaluate the effect of (−)-carveol on the HUA vascular musculature demonstrate its vasorelaxant effect, through the inhibition of L-type voltage-operated Ca^2+^ channels, as well as its partial involvement with large conductance Ca^2+^ activated K^+^ channels (BKCa). Further studies aiming to understand the possible mechanisms adjacent to those found in this study with (−)-carveol are thus proposed.

## 5. Conclusions

In conclusion, our results clearly demonstrate that (−)-carveol has an important vasorelaxant effect on the contractility of HUA smooth muscle cells, with its greater pharmacological potency in the pharmacomechanical coupling involving the serotonergic ECC standing out. Our study provides data that demonstrate the effective participation provided of VOCCs and a partial modulation of BKCa channels by (−)-carveol, suggesting that blockade of these channels may contribute to the relaxation produced by (−)-carveol. These unprecedented and innovative results with the natural monoterpene (−)-carveol suggest this compound may be a promising natural product in the vasorelaxant therapy of diseases that result in increased resistance in umbilical arteries, such as pre-eclampsia.
